# Supplementary material for: Content-rich biological network constructed by mining PubMed abstracts
Source: BMC Bioinformatics. 2004 Oct 8;5:147. doi: 10.1186/1471-2105-5-147 (PMC528731; doi:10.1186/1471-2105-5-147)
Supplement: Additional File 2 — The original results of the above study (non-essential files are deleted to keep the file size under the limit set by BMC bioinformatics). [file 1471-2105-5-147-S2.bz2 › chilibotAdditionalFile2/dip10/55ID8493578E210/html/ELF1_RB1.html]

 


 **ELF1** and **RB1** 
  
Found 5 abstracts in PubMed, retrieved 5.  
 

 What does Google say? 
 PDF only 
| .edu only 

---

**Interactive relationship** (e.g. stimulation, inhibition, etc)

**Neutral relationship**- The 98 kDa Elf 1  [ **ELF1** ]  is released from the cytoplasm tethering retinoblastoma  [ **RB1** ]  protein and moves to the nucleus, where it binds to the promoter of the TCR zeta chain gene.  Ref: 11884456 J Immunol, 2002

**Non-interactive relationship** (e.g. studied together, co-existance, homology, etc.)

- The present study reports structural similarities between viral oncoproteins, growth factors belonging to the insulin family, members of the steroid thyroid receptor superfamily, a D type cyclin, the Elf 1  [ **ELF1** ]  transcription factor and Bcl oncoproteins in regions that have been shown or proposed to mediate complex formation of these proteins with the tumor suppressor retinoblastoma  [ **RB1** ]  protein RB.  Ref: 7776898 Med Hypotheses, 1995
- Elf 1  [ **ELF1** ]  binds exclusively to the underphosphorylated form of Rb and fails to bind to Rb mutants derived from patients with retinoblastoma  [ **RB1** ] .  Ref: 8493578 Science, 1993
- Regulation of the Ets related transcription factor Elf 1  [ **ELF1** ]  by binding to the retinoblastoma  [ **RB1** ]  protein.  Ref: 8493578 Science, 1993
